# Novel Polyurethane-Based Systems Modified with Starch and Phase Change Materials for Bone Tissue Regeneration

**DOI:** 10.3390/polym15224414

**Published:** 2023-11-15

**Authors:** Klaudia Ordon, Piotr Szatkowski, Wojciech Piekarczyk, Elżbieta Pamuła, Kinga Pielichowska

**Affiliations:** Department of Biomaterials and Composites, Faculty of Materials Science and Ceramics, AGH University of Krakow, Al. Mickiewicza 30, 30-059 Krakow, Poland; klaudia.ordon@interia.eu (K.O.); pszatko@agh.edu.pl (P.S.); wpiekar@agh.edu.pl (W.P.); epamula@agh.edu.pl (E.P.)

**Keywords:** biomaterials, polyurethanes, starch, poly(ethylene glycol)

## Abstract

Novel polyurethane-based materials have been synthesized by a two-step process using poly(ε–caprolactone) diol (PCL) and 1,3–propanediol/starch (PDO/ST) systems as chain extenders/cross-linkers and 1,6–hexamethylane diisocyante (HDI) as a potential material for bone tissue replacement or bone cements. A poly(ethylene glycol)/starch (PEG/ST) system has been applied as a form-stable phase change material (PCM) to decrease the maximum setting temperature, while hydroxyapatite (HAp) has been used as a bioactive nanofiller. FTIR and SEM-EDX analyses were performed to investigate the structure, surface morphology, and thermal properties of the obtained polyurethanes. FTIR spectroscopy confirmed the chemical structure of the synthesized polyurethanes. SEM-EDX analysis confirmed the incorporation of starch/hydroxyapatite into the polyurethane matrix. Modification with PCMs based on PEG or PEG/starch systems allowed for a decrease in the maximum setting temperature of PUs from 6 to 7.6 °C, depending on the type of PCM used. Thus, the obtained polyurethanes show a good energy storage effect and a good application potential for the synthesis of multifunctional bioactive materials for future use as bone cements.

## 1. Introduction

Polyurethanes (PUs) are an attractive candidate in biomedical engineering because of the possibility of extensive chemical or physical modification to control their properties. Properly designed PUs are characterized by their non-toxicity, biocompatibility, controlled degradation rate, resistance to sterilization, stability of physical properties over time, and also promotion of calcination in vivo. The desired properties can be tailored to specific clinical applications by changing the chemical composition, raw material proportions, polymerization method, and synthesis or technology parameters [1]. However, based on an analysis of the literature in the area of bone cements, it can be concluded that little has changed over the years with regard to the composition of acrylate cements. As a consequence, the problem of high cement setting temperatures and the presence of a tissue-toxic monomer remain unresolved [2]. Today, efforts are being made to create a multifunctional bone cement that is bioactive and biodegradable, with appropriate wettability and a structure that stimulates the growth of new bone tissue. PUs are potential candidates for the reconstruction and regeneration of damaged or deformed tissues [3,4]. However, during polymerization and curing processes, a strong exothermal reaction also occurs, and to decrease the maximum setting temperature, bone cement can be modified with phase change materials that can efficiently decrease the maximum setting temperature [5].

Phase change materials (PCMs) are functional materials capable of exchanging thermal energy with their environment within a slight or no temperature change [6]. According to their phase change states, PCMs can be divided into two main groups: solid–liquid PCMs and solid–solid PCMs [6,7,8]. Among them, solid–solid phase change materials deserve special attention due to their small volume change, lack of phase separation, and lack of leakage [9,10].

Poly(ethylene glycol) (PEG) is one of the most studied PCMs, as it is characterized by adjustable phase change temperature, good biodegradability, low cost, and relatively high heat of fusion, which is attributed to a high degree of crystallinity [8]. The molar mass is an essential issue for the PCM application. An increase in the average molar mass of PEG contributes to an increase in the melting point from ca. 4 to 70 °C, with melting heat in the range of 117–174 J/g. However, the main problem with the application of PEG for latent heat storage is leakage above its melting temperature because of the solid–liquid phase transition. Liquid leakage from PCM can affect properties and features of the host material, such as a decrease in mechanical strength, an increase in flammability, or a colour change [8]. To avoid PCM leakage, several PEG stabilization methods have been described in the literature, such as shape stabilization with inorganic materials, for example, cross-linking of silica [11,12,13], encapsulation [14,15], cross-linking with the use of polysaccharides [10,16], or PU formation [8,17,18,19]. Pielichowska and Pielichowski prepared biodegradable poly(ethylene oxide) (PEO)/potato starch (ST) blends as solid–solid PCMs. They found that the presence of ST changed the PEO phase-transition behaviour and that there is a powerful intermolecular interaction between PEO and ST. For PEO/ST 1:3 and 1:1 *w*/*w*, a solid–solid phase transition was observed [10]. In turn, Harle et al. [8] prepared linear and cross-linked-shape-stabilized PCMs based on PEG with different molecular weights via one-step bulk polymerization. Their results indicated a solid–solid phase transition, good thermal stability, high transition enthalpy, and a suitable transition temperature.

Among solid PCMs, ST is receiving increased attention because of its complete biodegradability in a wide variety of environments, ease of functional property optimization, availability, renewability, and low cost. ST can be hydrolysed by microorganisms or enzymes into glucose and subsequently metabolized into carbon dioxide and water [8]. The chemical and physical properties of ST are determined by its structure and composition. ST is a polysaccharide polymer consisting of repeating 1,4–α–D–glucopyranosyl units. ST is a material containing two types of structures: linear and branched. The linear component, amylose, with an average molecular weight of approximately 1 × 10^6^ g/mol, represents the amorphous structure of starch and is the minor component (20–30%). In turn, the branched component, amylopectin, with an approximate molecular weight of 1 × 10^8^ g/mol, represents the crystalline structure. The branching of amylopectin creates double helices packed in crystallites. Modification of ST is necessary to achieve the required properties [8,20,21,22]. Many methods of modification of ST granules involve their granular disruption to access –OH functional groups [20].

The presence of hydroxyl groups in ST enables a chemical reaction with diisocyanates or urethane prepolymers [23]. The combination of these groups of polymers allows us to obtain a whole range of new materials with desirable properties for orthopaedic applications. The use of ST with multiple hydroxyl groups leads to the formation of a three-dimensional network of covalent bonds, which helps increase the mechanical compressive strength of PUs [24]. This has a direct impact on the final properties of PU, such as dimensional stability, tensile strength, elastic modulus, and hardness [25]. The addition of starch to PU improves its biodegradability and mechanical properties, providing a filler and cross-linking effect. Javaid et al. [21] obtained PU modified with starch synthesized by the reaction of isophorone diisocyanate and hydroxyl-terminated polybutadiene, which was extended to include different molar ratios of 1,4–butanediol (BDO) and corn starch. Studies have shown that ST is a better chain extender than conventional diol. ST-based PU showed a higher molar mass than BDO-based PU.

HAp is the bioactive ceramic that is most similar to the mineral part of bone and tooth enamel and enhances bone cell attachment, proliferation, and migration. Soluble and/or nanosized calcium phosphates were also reported to exhibit osteoinductive properties, thus actively promoting the growth and regeneration of new bone. HAp has been widely studied in bone repair and regeneration, especially as a filler in polymer composites and metallic implant coatings to improve the interaction with hard tissue and implant bioactivity [26,27,28].

This article reports on the results of the synthesis and characterization of novel PU-based systems as potential bone cements for orthopaedic applications modified with a solid–liquid or solid–solid phase change material to decrease the maximum setting temperature. The PUs were also modified with hydroxyapatite (HAp) to improve their bioactivity. Starch was used both as a PU cross-linker and as a shape stabilizer for PEG-based PCMs. The effects of PCMs on chemical structure, morphology, mechanical properties, wettability, bioactivity, in vitro chemical response, and MC3T3 preosteoblastic cell response were studied [29].

## 2. Materials and Methods

### 2.1. Materials

Poly(ε–caprolactone) diol (PCL, M_n_ = 2000 g/mol), 1,6–hexamethylene diisocyanate (HDI), dibutyltin dilaurate (DBTDL), 1,3–propanediol (PDO), and PEG with a 4000, 6000, 8000, and 10,000 average molar mass were purchased from Sigma-Aldrich (St. Louis, MO, USA). Potato starch (ST) was provided by Chempur (Piekary Śląskie, Poland). HAp needle-like nanopowder (60 nm) was supplied by mkNano (Mississauga, ON, Canada). Polyol (PCL) and PEG were dried at 90 °C in a vacuum for 2 h prior to synthesis to remove air and moisture. In turn, ST and HAp were dried under vacuum at 100 °C for 24 h before use. Diisocyanates and all other reagents were analytical grade and were used as received.

### 2.2. Synthesis of Polyurethanes

Polyurethane materials were prepared by a two-step bulk polymerization process according to our previous work [24]. According to this procedure, in the first step, PCL and HDI were reacted to obtain the -NCO-terminated PU prepolymer, keeping the mole ratio at 1:3. For this purpose, 1 mol of PCL was introduced into a preheated, three-necked glass flask equipped with a heating bowl, thermometer, mechanical stirrer, reflux condenser, drop funnel, and nitrogen supply nozzle. Subsequently, DBTDL catalyst and 3 mol of HDI were added. The reaction was carried out for 1 h at 60 °C. In the second stage, a chain extension/cure process was performed. In this step, the ultimate PUs were obtained by adding chain extender/cross-linker (CE, PDO/ST mass ratio 1.5/0.5), HAp, and PCM to the reaction mixture, according to Table 1. After homogenization, the PU samples were heated for 24 h at 80 °C [21,24].

### 2.3. PCMs Shape Stabilization

In the preparation procedure, shape-stabilized PCM (PEG/ST), PEG400 or PEG8000, and ST were initially dissolved/dispersed in distilled water, maintaining a total solid concentration of 3 wt.% with PEG:ST in different ratios of 90:10, 85:15, 80:20, and 75:25. In the next step, the mixtures were heated to the gelling point (ca. 72 °C) using a water bath and kept for 15 min at this temperature. The PEG/ST/water gels were then cast onto Petri dishes and dried at room temperature for several days to remove water.

### 2.4. Techniques

FTIR spectra were recorded with a Tensor 27 spectrometer from Bruker (Bruker, Billerica, MA, USA), which was equipped with a diamond ATR crystal. For each spectrum, at room temperature in a range of 4000–600 cm^−1^, 64 scans with a resolution of 4 cm^−1^ were collected.

On the basis of the FTIR spectra, the degree of phase segregation (DPS) of PU samples was determined according to the equation:

DPS = R/1 + R(1)

For this purpose, the hydrogen bond index (R) was determined:

R = A_B_/A_F_ (2)

where A_B_—area of the absorption band from the C=O urethane group bound by a hydrogen bond and A_F_—area of the absorption band from the urethane carbonyl group not hydrogen bonded.

The R value in hard segments determines the ratio of the share of C=O urethane groups connected by hydrogen bonds to the share of analogous groups not bound by hydrogen bonds. R was determined from the absorption bands in the range of 1700–1712 cm^−1^, originating from the stretching vibrations of the free carbonyl group in the urethane bond, and in the range of 1683–1686 cm^−1^, originating from the vibrations of the hydrogen-bonded C=O group. The band values in the given wavenumber ranges were obtained as a result of deconvolution of the bands originating from the vibrations of the carbonyl groups obtained for PU into component bands (Gaussian distribution) using OPUS Spectroscopy Software 8.5. 

Wide-angle X-ray diffraction (WAXD) studies were carried out using the X’Pert Pro diffractometer from Panalytical (Malvern, UK) using monochromatic CuKα radiation (λ = 1.5406 Ǻ, 40 kV, 40 mA) obtained using a germanium monochromator (Ge (111). Measurements were carried out at room temperature in Bragg–Brentano geometry. Data were recorded in the 2θ range from 5° to 60° with a resolution of 0.001°.

Scanning electron microscopy (SEM, Nova NanoSEM 200, FEI, Eindhoven, The Netherlands) equipped with an energy dispersive X-ray (EDX) analyser (EDAX Company, Mahwah, NJ, USA) at 5 kV electron beam energy with magnification of 500–10,000× and a working distance of 6.8–6.8 mm was used to study PUs microstructure. The surfaces of the samples for SEM were prepared by breaking them in liquid nitrogen. SEM observations were made after the prepared samples were sputtered with a thin layer of carbon. 

DSC measurements were performed using a Mettler Toledo DSC1 calorimeter (Greifensee, Switzerland); the sample weight was ca. 4 mg. The samples were placed in pierced and sealed aluminium pans. The DSC curves were recorded at a heating rate of 10 K/min over the temperature range of 37–150 °C in a nitrogen atmosphere (30 mL/min).

The mechanical properties of the obtained PU were tested using a Zwick 1435 (ZwickRoell GmbH & Co. KG, Ulm, Germany) test machine in a static compression test. Cylindrical samples with dimensions of 12 mm × 10 mm were tested. Measurements were carried out at room temperature with a compression speed of 2 mm/min. The compression test was carried out on up to 50% of the sample deformation. The results were analysed using TestXpert III software. Young’s modulus (E) and compressive strength (σ_m_) were determined from the graphs of force versus deformation of polyurethanes. The results obtained were the average of three measurements for each sample.

The elastic properties of the samples were characterized using the ultrasonic method. The tested samples had the shape of cylinders with dimensions of height (h) from 14 to 19 mm and diameter (Φ) from 18 to 20 mm. Measurements of the propagation velocity of longitudinal ultrasonic waves were carried out using a Unipan Ultrasonic–CT3 materials tester with a pair of low-frequency ultrasonic heads (f = 1 MHz) and a head diameter of 20 mm, using the pass-through method. Insulating tape patches were used as the coupling medium. Other measurement parameters of the apparatus were as follows: energy (amplitude of the transmitting pulse): 600 V, gain: +20 dB, and repetition frequency: 1 Hz. 

In vitro chemical stability tests of PU materials were carried out according to the ISO 10933 standard [30] by incubating samples with dimensions of 12 mm × 2 mm in phosphate-buffered saline (PBS) at pH = 7.4 and in Ringer solution at pH = 7. The ratio of sample mass [g] to solution volume [mL] was 1:100. Incubation was carried out at 37 °C for 12 weeks. The pH of the solutions was measured once a week.

The wettability of the PU samples was tested using the Krüss DSA25 drop shape analyser (Kruss, Hamburg, Germany). The contact angle (θ) of the surface was determined using distilled water through measurements made at room temperature for ten drops with a volume of approximately 1 µL using the sitting drop method. 

The bioactivity of the materials was tested by incubating them in simulated body fluid (SBF) with an ionic composition similar to human plasma for 14 and 28 days at a temperature of 37 °C [31]. The ratio of sample mass [g] to solution volume [ml] was 1:100. The SBF was replaced every three days during the incubation period. To confirm the formation of an apatite layer on the surface of the material after incubation, the samples were removed from the SBF, washed with distilled water, dried, and analysed using SEM/EDX microscopy.

Cytotoxicity tests were performed using extracts from selected PU/PCM systems according to the international standard ISO 10993-5 [32]. The 10% wt/vol extracts were prepared by incubation of the samples in cell culture medium (DMEM, PAN BIOTECH, Aidenbach, Germany) for 24 h at 37 °C with the addition of 10% bovine serum (foetal bovine serum, Biowest, Nuaillé, France) and 1% antibiotics (penicillin/streptomycin, PAA, Leonding, Austria). 

Before being added to the cells growing on the bottom of the wells, the extracts were sterilized by filtration using syringe filters (0.22 µm). Extracts in the following dilutions were used in the studies: 100% (undiluted extract), 50%, 25%, 12.50%, and 6.25%. A culture medium was used to dilute the extract, and a non-diluted medium was used as a reference sample.

The selected PU/PCM systems were tested in contact with the MC3T3 preosteoblastic cells (MC3T3–E1 subclone 4, ATCC, Teddington, UK). Cells were cultured in the DMEM medium (PAN BIOTECH, Aidenbach, Germany) with the addition of 10% bovine serum (foetal bovine serum, Biowest, Nuaillé, France) and 1% antibiotics (penicillin/streptomycin, PAA, Leonding, Austria). Culture was carried out at 37 °C, 5% CO_2_, and increased humidity. Cells were seeded in 96-well plates at 5000 cells per well in 100 µL of medium. After 24 h of incubation, the medium above the cells was replaced with undiluted and diluted extracts (100 µL). After another 24 h, the metabolic activity of the cells and their viability were examined using the AlamarBlue test and live/dead staining. The tests were performed using a FluoStar Omega spectrofluorimeter from BMG LABTECH and a Zeiss Axiovert 40 fluorescence microscope (Carl Zeiss, Aalen, Germany), respectively. The AlamarBlue test allowed for the calculation of the percentage reduction of resazurin based on the formula:

Resazurin reduction (%) = (F_sample_ − F_0% red_/F_100% red_ − F_0% red_)·100% (3)

where F_sample_—fluorescence of the sample, F_0%red_—fluorescence of the culture medium with the addition of AlamarBlue reagent without cells, and F_100% red_—fluorescence of the culture medium with the addition of AlamarBlue reagent reduced by 100% by autoclaving (15 min at 121 °C). The results of the metabolic activity test (AlamarBlue) were reported as the mean ± standard deviation of three independent measurements (*n* = 3).

## 3. Results

**Figure 1:**
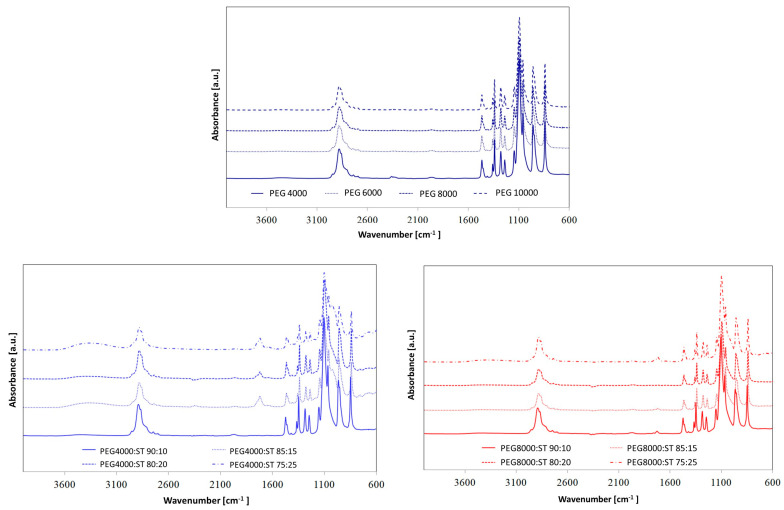
FTIR spectra of PCM based on PEG with different average molar masses, PEG4000/ST and PEG8000/ST.

**Figure 2:**
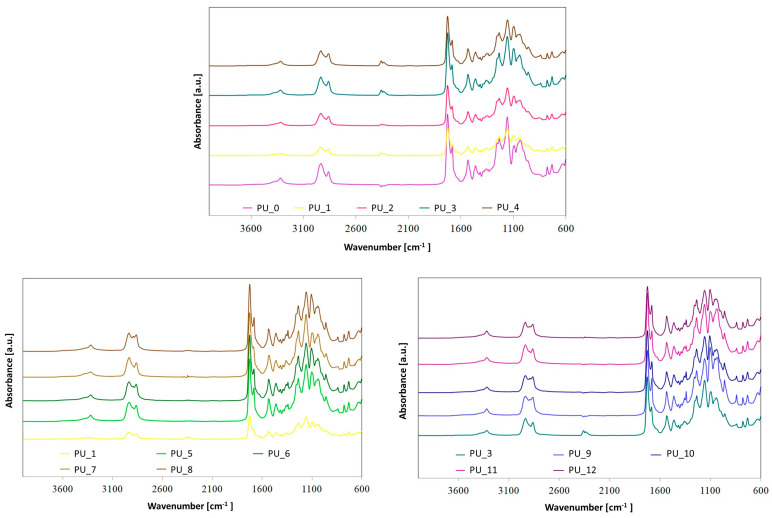
FTIR spectra of the obtained PUs with the addition of PCM based on PEG or PEG/ST PCMs.

In order to assess the chemical structures of synthesized PUs, FTIR spectroscopy (Figure 1 and Figure 2) was used.

The description of characteristic absorption bands for PUs is given in Table 2. 

The absorption bands at 3316–3333 cm^−1^ are associated with the stretching vibration of –NH in the urethane moieties. These groups are involved in the hard segment-soft segment (HS–SS) and hard segment-hard segment (HS–HS) H-bond formation. All spectra also show absorption bands in the range of 2937–2936 cm^−1^ and 2863–2858 cm^−1^, which are related to the asymmetric –CH_2_ and symmetric –CH_2_ stretching vibrations, respectively. The characteristic peaks located at ca. 1733–1680 cm^−1^ are connected to the stretching vibrations of carbonyl groups in urethane bonds. The bands in the range of 1733–1726 cm^−1^ are connected to free C=O, while peaks near 1684–1682 cm^−1^ correspond to hydrogen-bonded C=O in HS–HS in urethane moieties. The absorption peaks at ca. 1538 cm^−1^ correspond to the deformation vibration of –NH bonds in the urethane groups. In turn, the absorbance in the range of 1145–1061 cm^−1^ can be assigned to the asymmetric stretching vibrations of the C–O–C group in PEG. Furthermore, no absorption bands were found in the range of 2200–2300 cm^−1^, indicating that all the –NCO groups reacted. These results show that the –NHCOO– bond has been formed in the PUs under investigation [33,34,35]. Furthermore, the lack of an absorption band originating from –OH groups in HAp at 3571 cm^−1^ confirms that HAp is covalently bonded to polymer chains. The presence of HAp in the synthesized PUs was verified by peaks at 1023 cm^−1^ (PO_4_^3−^) and 1427 cm^−1^ (CO_3_^2−^) [35].

By comparing the FTIR spectra of the PUs, it can be concluded that the relative positions of the different absorption bands have not changed significantly, which indicates that the presence of different PCMs has a slight effect on hydrogen bonding in PU systems [34].

**Figure 3:**
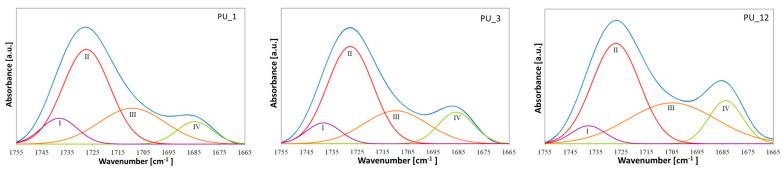
Band from stretching vibrations of the C=O group after deconvolution for selected samples of PU modified with PEG-based PCM.

However, special attention should be paid to the areas originating from the stretching vibrations of C=O groups, because they are particularly important in PU phase separation. The characteristic bands of the carbonyl group appear in the range of 1686 to 1740 cm^−1^. The band at approximately 1708 cm^−1^ corresponds to the non-hydrogen-bonded carbonyl groups in the urethane groups, and in the range from 1683 to 1686 cm^−1^ comes from the hydrogen-bonded C=O group of the urethane moiety [36]. The position of these bands shifts to slightly higher wavenumbers after introducing different shape-stabilized PCMs, which may indicate a weakening of hydrogen bonds [37]. This is also confirmed by the calculated hydrogen bond index (R) and degree of phase separation (DPS)—see Figure 3 and Table 3.

PEG-based PCMs with different average molar masses hindered the formation of HS–HS hydrogen bonds in PU, which led to greater phase mixing. In turn, the addition of PCM based on PEG4000/ST or PEG8000/ST, compared to the PU_1 and PU_3 samples, respectively, resulted in an increase in the number of hydrogen bonds in the PUs produced. The degree of phase separation of PU with the addition of PEG8000/ST is higher than that of PU modified with PEG4000/ST, suggesting that the introduction of the PEG8000/ST system promotes the formation of hydrogen bonds and phase segregation [38]. 

**Figure 4:**
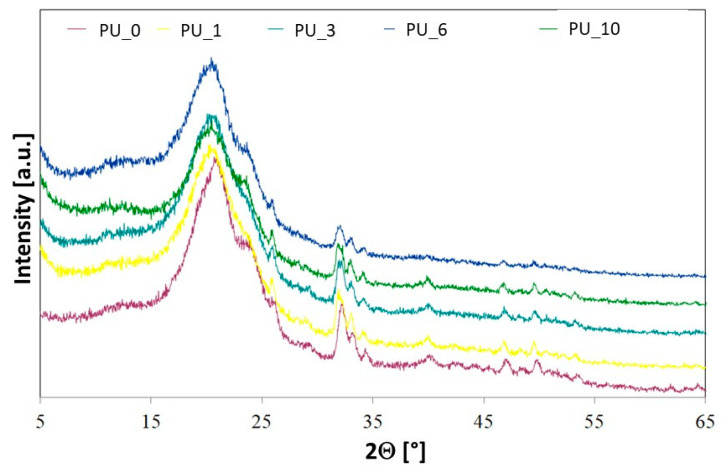
XRD spectra for selected PU/PCM systems.

Crystallinity in selected PU/PCM systems was examined using wide-angle X-ray diffractometry (WAXD) (Figure 4). The crystallinity of polyurethanes is largely ensured by soft and hard segments [39]. PCL is a semicrystalline thermoplastic polymer with characteristic reflexions in the XRD spectra located at 21.5°, 22°, and 23.7°. The results obtained for PCL are consistent with the XRD results reported in the literature [40]. The results obtained show that the crystallinity of the tested PUs was much lower than that of the starting material, which suggests that the soft PU segments did not crystallize due to insufficient segment mobility. This may also be due to the strong interaction of the urethane bond with the hydroxyl groups of polyol, polysaccharide, or HAp [41]. The increase in PU cross-linking additionally limits crystallization and reduces the crystallinity of the tested PUs [42].

For PEG, characteristic reflexions are observed at 19.2° and 23.3° [43]. After the PEG-based PCM or PEG/ST system was added to the PU matrix, no additional reflections were observed in the XRD spectra. The profiles of the characteristic reflections of the tested PU/PCM systems were similar to those of the PU_0 sample, which indicates that the addition of PCM did not have a major impact on the interplanar spacing [43]. However, the influence of the addition of PCM on the intensity of characteristic reflections of the tested materials is visible, which decreased slightly after the introduction of PEG-based PCM. The ability to crystallize depends on the chain mobility, leading to the conclusion that in PU, the addition of a PEG-based PCM or PEG/ST system decreases the mobility of the segments, which reduces crystallinity and makes PU a more amorphous material [43].

**Figure 5:**
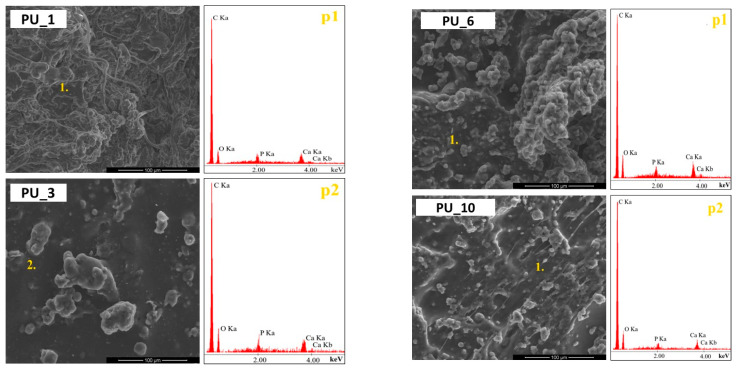
SEM microphotographs of fracture surfaces of PUs with PEG4000, PEG8000, PEG4000/ST (85:15), and PEG8000/ST (85:15), as well as results of EDS analysis at points 1 and 2.

Surface analysis of the prepared polyurethane samples using PCMs with different molar masses of PEG (PU_0–PU_4) was performed using SEM analysis, as shown in Figure 5. 

It was observed that on the surfaces of the PUs analysed, the starch grains and HAp particles were completely covered by the PU layer, indicating a strong bond between the introduced additives and the PU matrix. Furthermore, the PUs were characterized by a relatively smooth surface and a good dispersion of the additives introduced, indicating the cohesive three-dimensional network structure of the PU obtained. In turn, the introduction into PU of gelatinized starch molecules from PEG with an average molar mass of 8000 (Figure 5) led to an uprising of a more uneven surface morphology compared to the PU_3 sample. The gelatinized starch molecules appeared as aggregates and were randomly dispersed throughout the PU layer. The SEM observations and the EDS results confirm the presence of starch and HAp in the prepared PUs. 

**Figure 6:**
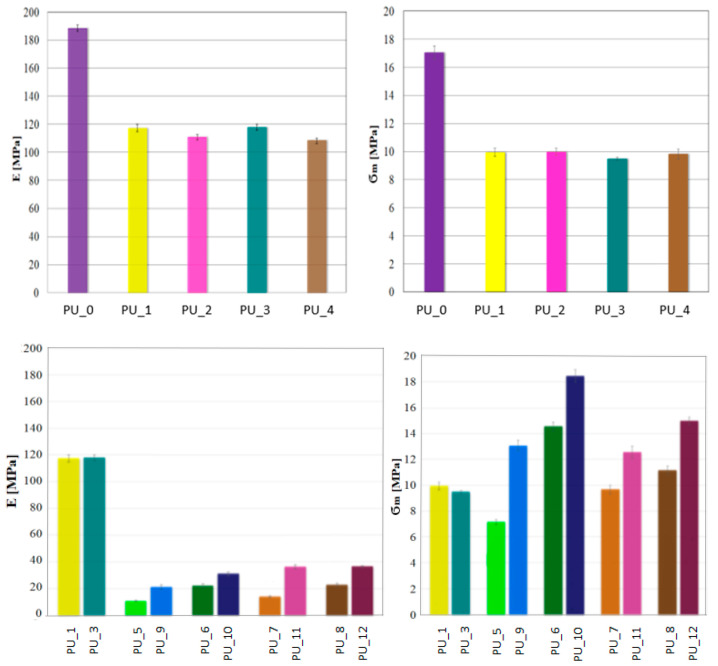
Results of the static compression test for PU with the addition of PCMs: Young’s modulus (E) and compressive strength (σ_m_).

Considering the potential use of PU with the addition of PCM, it is crucial to determine their mechanical properties. A classic uniaxial compression test was performed to determine the compressive strength and Young’s modulus of PCM-modified PUs. The values for the average compressive strength and Young’s modulus for the PU/PEG system with different average molecular weights and for PUs modified with PEG4000/ST or PEG8000/ST are shown in Figure 6. 

The research shows that the addition of PCM in the form of PEG with a different average molar mass reduces the compressive strength of the tested materials. There was no effect of the average molecular weight of PEG on the mechanical parameters. The decrease in compressive strength and Young’s modulus is most likely related to the slowing of the polymerization reaction or to the phase transition of PEG during the polymerization of PU.

The porous structure promotes cell proliferation in the process of bone tissue formation, as a result of which the ultimate mechanical strength of PU would increase. The addition of PCM in the form of a PEG/ST system improved the compressive strength of PU. It can be concluded that PEG leakage due to the solid–liquid phase transition has been eliminated or significantly reduced. The PU_10 sample has the highest compressive strength. 

**Figure 7:**
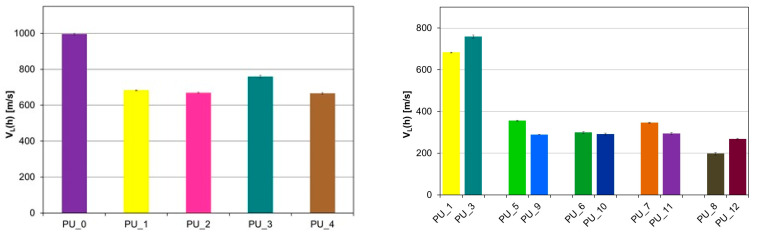
Values of the velocity of the longitudinal ultrasonic wave at height in PU modified with PCM based on PEG with different average molar masses (**left**) and PEG/ST (**right**).

**Figure 8:**
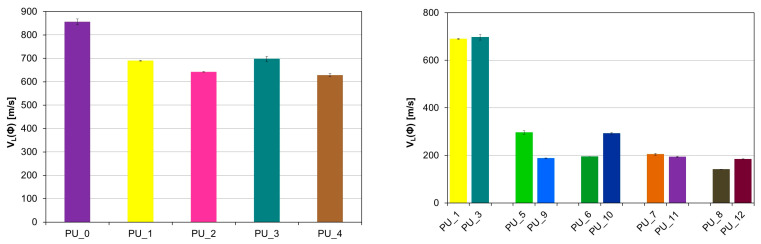
Values of the velocity of the longitudinal ultrasonic wave along the diameter in PU with the addition of PCM based on PEG with different average molecular weights (**left**) and PEG/ST (**right**).

A significant decrease in the velocity of the longitudinal ultrasonic wave (v_L_) was observed compared to the PU/starch and PU/HAp systems for PU with the addition of PCM (Figure 7 and Figure 8).

For PU with the addition of PCM based on PEG4000/ST, the average value v_L_ (h) was 299.3 m/s, while for PU with the addition of PCM based on PEG8000/ST, the average value v_L_ (h) was 285.2 m/s s (V). This indicates a significant reduction in the mechanical properties of these samples as a result of the increase in the number of defects in the structure. PUs modified with PEG/starch blends are less homogeneous than unmodified PCM PU or PEG-modified PU with an appropriate average molecular weight. This is consistent with the morphological characteristics of the materials tested by SEM. The results obtained from diameter measurements also confirm a significant reduction in the velocity v_L_. The values of the anisotropy A of longitudinal ultrasonic wave velocity were determined using the following formula: A = [(v_L_ (h) − v_L_ (Φ)/v_L_ (h)]·100% range from 4% for PU with the addition of PCM based on PEG with different average molar masses to 30% for PU with the addition of PCM based on PEG4000/ST or PEG8000/ST. To characterize the elastic properties of an anisotropic material with transverse isotropic symmetry, it would be necessary to determine five independent elastic constants or seven material constants [44], and it is not possible to determine the velocities of the shear ultrasonic waves with the appropriate polarization due to the very high attenuation of the ultrasonic wave. Therefore, the values of Young’s modulus (E) were estimated based on the results obtained v_L_ (h) at height, the assumed Poisson’s ratio μ = 0.35, and the determined value of apparent density.

**Figure 9:**
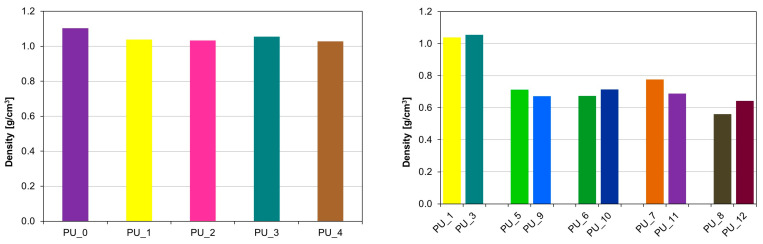
Apparent density of the tested PUs modified with PCM based on PEG with different average molar masses (**left**) and PEG4000/ST or PEG8000/ST (**right**).

Figure 9 shows the apparent density values of the tested samples. Modification of PEG/ST with different molar masses of 4000 and 8000 g/mol, used as PCM to store the thermal energy released during cross-linking of PU, caused a decrease in apparent density to 60% from the average value of 1.10 g/cm^3^ for PU/HAp up to 0.68 g/cm^3^ for PU with the addition of PCM based on PEG and starch.

**Figure 10:**
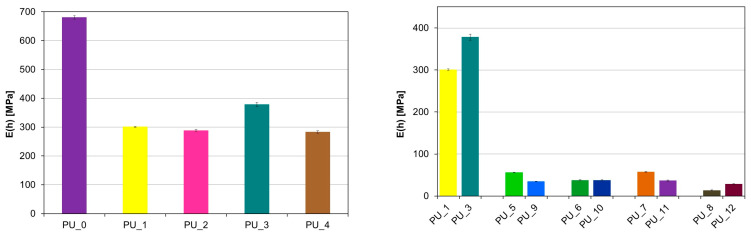
Young’s modulus values—height testing of PU samples modified with PCM based on PEG with different average molar masses (**left**) and PEG4000/ST or PEG8000/ST (**right**).

Based on the above results, the theoretical Young’s modulus was calculated, and its values are presented in Figure 10. The PU_3 sample is characterized by the highest values of the Young’s modulus. The introduction of starch-modified PCM resulted in a significant reduction in the E value. The results obtained are consistent with those obtained during uniaxial compression of the materials.

**Figure 11:**
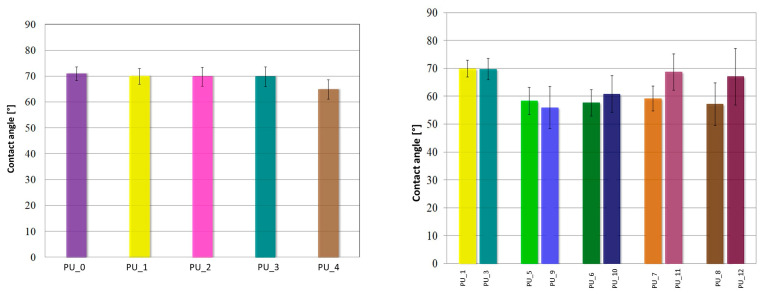
Contact angle values for PU modified with PEG-based PCM with different average molar masses (**left**) and PEG4000/ST or PEG8000/ST (**right**).

The contact angle measurement results for PU/PCM systems are shown in Figure 11.

PUs do not show significant changes in wettability in the presence of PEG-based PCM with different average molar masses. For unmodified PCM PU (PU_0), the contact angle is 70.1 ± 2.6° and is comparable to other PU/PCM systems, where the contact angle ranges from 70 ± 3.5° for PU_1, PU_2, and PU_3 and up to 65 ± 3.7° for PU_4. These results show that the modification with PEG did not affect the surface properties of PU. PU, with the addition of PCM based on PEG4000/ST or PEG8000/ST, was characterized by an increase in surface hydrophilicity. For samples with the addition of PCM based on PEG4000/ST, the average value of the contact angle was 58.4 ± 5.4°, while for PU with the addition of PEG8000/ST, it was θ = 67.2 ± 7.7°. In summary, the presence of PCM based on the PEG/ST system changes the wettability of PU, thus increasing its hydrophilicity, which is a desirable phenomenon for biomaterials intended to interact with bone tissue. 

**Figure 12:**
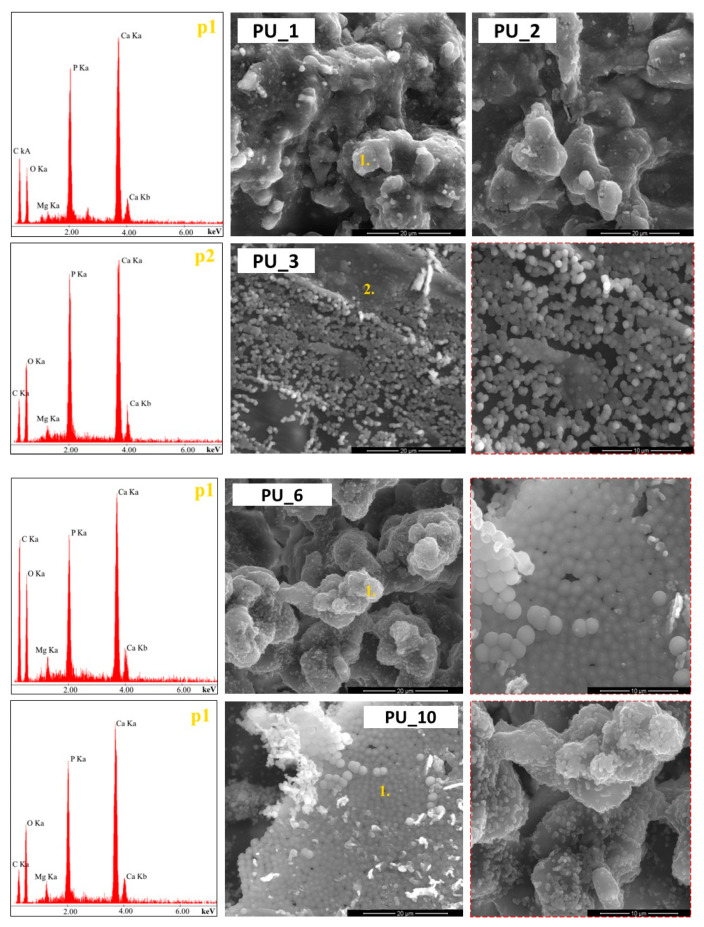
SEM microphotographs and EDX analysis results at point 1 for PU with the addition of PEG4000/ST or PEG8000/ST after a two-week incubation in SBF.

The preliminary assessment of bioactivity showed the presence of apatite deposits on the surfaces of all PU/PCM systems after incubation in SBF (Figure 12). Depending on the type of PCM used to modify PU, different rates of apatite growth were observed. As the molar mass of PEG increased and the incubation period increased, the formation of more compact calcium phosphate monolayers was observed.

The addition of PEG4000/ST or PEG8000/ST accelerated the crystallization of the apatite layer on the surface of the materials. The appearance of a larger apatite layer may also be influenced by the microstructure or porosity of the PU itself. After a four-week incubation, compact apatite layers are observed, especially on samples containing more than 15 wt% starch in PCM. The appearance of compact apatite layers on PU surfaces with the addition of PEG/ST indicates the potential ability of the discussed materials to create a permanent bond with bone tissue.

**Figure 13:**
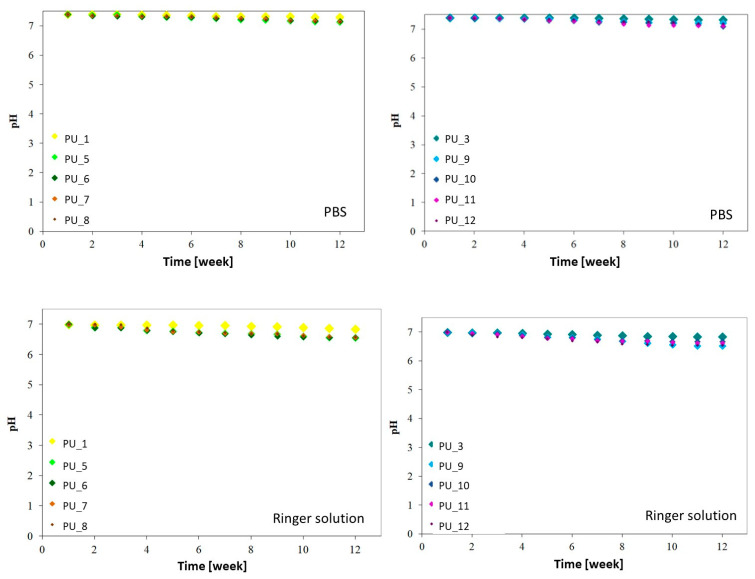
Change in pH of PBS and Ringer’s solution as a function of incubation time for PU with the addition of PCM based on PEG with different average molar masses and PEG4000/ST or PEG8000/ST.

The in vitro chemical stability of PUs was evaluated with the addition of PCM based on the changes occurring during incubation in PBS and Ringer’s solution (Figure 13).

The results obtained show that the degradation of PU with the addition of PCM based on PEG with different average molar masses and PEG4000/ST or PEG8000/ST takes place without major pH fluctuations. Regardless of the environment in which all tested materials are incubated, the pH value slightly decreases throughout the incubation period and is in the range of 7.4 to 7.1 for PBS and 7.0 to 6.52 for Ringer solution. The presence of PEG4000/ST or PEG8000/ST accelerates the degradation process of the tested PUs, but its nature is gradual and still relatively slow. The decrease in PU pH of PEG-/ST-modified systems may be related to starch degradation as a result of the hydrolysis reaction or the dissolution of PEG. Another factor determining the faster degradation process may be the development of the specific surface area of the tested PUs. Nevertheless, the obtained results indicate that the obtained PU materials can be used to obtain biodegradable scaffolds.

**Figure 14:**
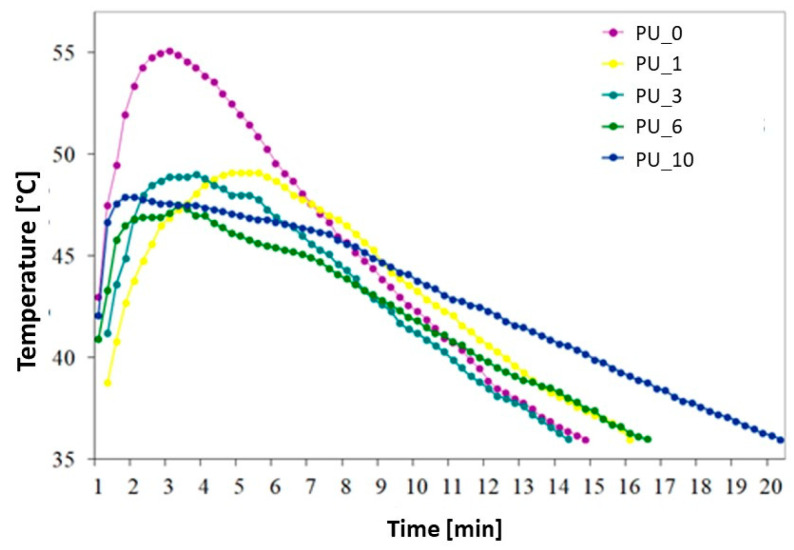
Temperature profiles of the curing process of selected PU/PCM systems.

The influence of the content of PEG or PEG/ST PCMs on the polymerization time and temperature of the obtained PU is shown in Figure 14 and Table 4. 

The maximum curing temperature (T_max_) of unmodified PCM PU was 55.1 °C. A decrease in T_max_ of PUs modified with PCM was observed from 6.0 to 7.6 °C, depending on the type of PCM used. No clear relationship was observed between the molar mass of PEG and the T_max_ of the PU curing process. The PEG/ST system significantly reduced the PU polymerization temperature and extended the polymerization time. The greatest temperature drop was recorded for PU with the addition of PCM based on PEG8000/ST. For this composite, a decrease in T_max_ by 7.6 °C and an extension of the polymerization time by 5 min and 30 s were found. The heat released during the polymerization reaction is partially absorbed by the endothermic effect of the phase transition of the PEG/ST system, thus leading to a reduction in the T_max_ of PU cross-linking. Although the PCM-added PU had a longer or shorter polymerization time than the unmodified PU, it still complies with the ISO 5833:2002 standard [45].

**Figure 15:**
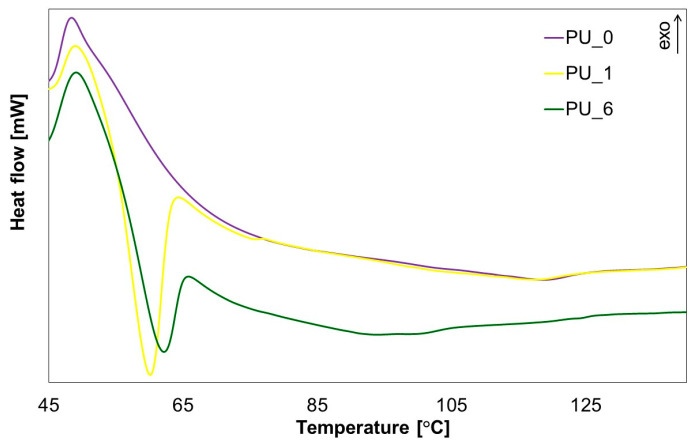
DSC curves of the PU polymerization process in the presence of selected PCMs.

To obtain more information about the cross-linking process of the tested PUs, selected materials with the addition of PCM were subjected to DSC analysis (Figure 15).

From the results obtained, it can be concluded that during PU cross-linking, the material undergoes a phase transformation by absorbing the heat of the polymerization reaction, which prevents excessive temperature increases within the system. This confirms that PCMs are capable of accumulating thermal energy in the form of latent phase change heat, which helps reduce the maximum cross-linking temperature of PU systems.

**Figure 16:**
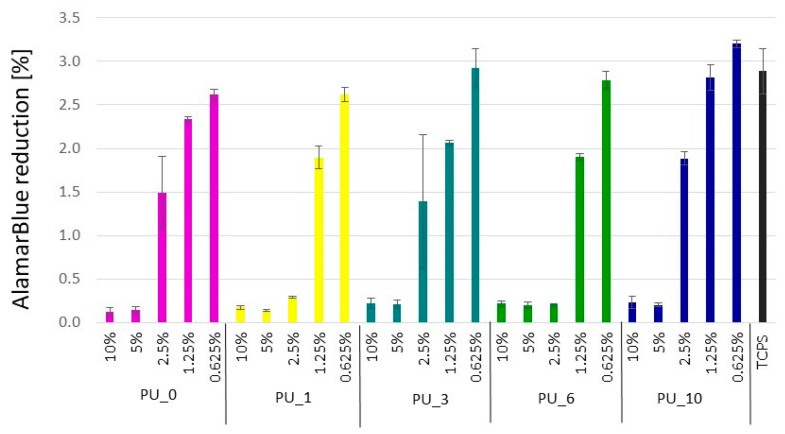
Metabolic activity of cells cultured for 24 h in the presence of 10%, 5%, 2.5%, 1.25%, and 0.625% extracts from selected PU/PCM systems.

**Figure 17:**
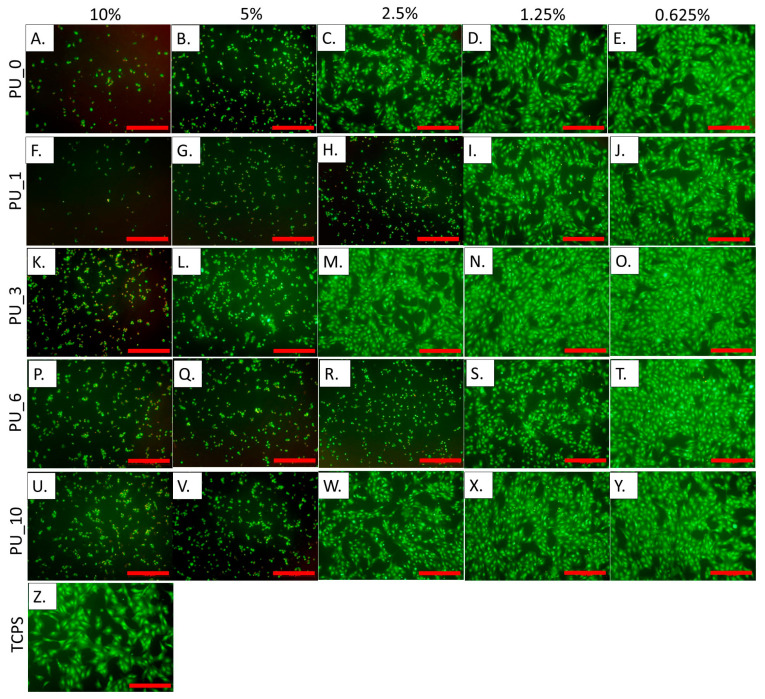
Live/dead staining of cells cultured for 24 h in the presence of extracts in the presence of 10%, 5%, 2.5%, 1.25%, and 0.625% extracts from selected PU/PCM systems. Scale bar = 100 µm.

Figure 16 shows the results of measuring the metabolic activity of cells incubated for 24 h in the presence of extracts from selected PU/PCM without dilutions, that is, 10% (undiluted extract), and diluted twice, four times, eight times, and 16 times, that is, 5%, 2.5%, 1.25%, and 0.625%, respectively. These results were confirmed by live/dead fluorescence staining. The representative microscopic images of cells are shown in Figure 17.

The study carried out in MC3T3 cells showed that undiluted 10% extracts cause cell death, and, moreover, the presence of dead cells was also visible with further dilutions. According to the standards, cell viability should be 70% compared to the control (medium). This value was achieved for all 0.625% extracts from the studied samples. PU_10 was found to be the most cytocompatible because cell viability was the same as that of the control when the extract concentration was 1.25%. The results indicate that the addition of PCM based on the PEG8000/ST system improves the cytocompatibility of the PU materials, which supports cell adhesion and proliferation. The live/dead test coincides well with the AlamarBlue analysis.

## 4. Conclusions

Polyurethane-based materials modified with PCMs were obtained by applying a two-step bulk polymerization method. PEG with different molar mases and PEG/ST were used as PCM. The FTIR-ATR analysis confirmed the PU structure. Microscopic observation results indicated quite good dispersion of HAp and ST and strong interactions between molecules. The chemical bonding of HAp and starch with PU macromolecules and the structure of the obtained PU were confirmed by spectroscopic tests. 

The XRD results show that the crystallinity of the tested PUs was much lower than the crystallinity of the starting PCL, which may be due to the difficulty in the crystallization of PCL after binding to PU macromolecules. The addition of PCM based on PEG or PEG/starch systems contributed to a decrease in the crystallinity of PU. The temperature profiles of the cross-linking process show a decrease in the T_max_ of PCM-modified PUs from 6 °C to 7.6 °C, depending on the type of PCM used. No clear relationship was observed between the molecular weight of PEG and the T_max_ of PU polymerization. The PEG/ST mixture significantly reduced the PU temperature and extended the polymerization time. The addition of PCM reduced the compressive strength of the modified PUs due to the melting of PEG during the PU polymerization reaction. The presence of PCM based on PEG and starch changed the wettability of PU, increasing its hydrophilicity, which is a desirable phenomenon for biomaterials. The tested materials showed good chemical stability in vitro and bioactivity, according to the SBF test. The study carried out in MC3T3 cells showed that undiluted extracts cause cell death. The results obtained showed that the addition of PCM based on a mixture of PEG 8000 and starch improves the cytocompatibility of the tested PU materials, supporting cell adhesion and proliferation. As a result of the wide possibility of modifying the chemical structure and physicochemical properties, polyurethanes may constitute a promising group of polymer materials for applications in the field of regenerative medicine.

## Figures and Tables

**Table 1:** Samples description.

Sample Name	PCL/HDI/CE Molar Ratio	PDO/ST Mass Ratio	Hap wt.%	PCM wt.%	Composition of PCM
PU_0	1:3:2	1.5:0.5	5	-	-
PU_1				15	PEG_4000_
PU_2					PEG_6000_
PU_3					PEG_8000_
PU_4					PEG_10000_
PU_5					PEG_4000_:ST 90:10
PU_6					PEG_4000_:ST 85:15
PU_7					PEG_4000_:ST 80:20
PU_8					PEG_4000_:ST 75:25
PU_9					PEG_8000_:ST 90:10
PU_10					PEG_8000_:ST 85:15
PU_11					PEG_8000_:ST 80:20
PU_12					PEG_8000_:ST 75:25

**Table 2:** Description of characteristic bands for PUR with PEG/ST different ratios.

	Wavenumber [cm^−1^]												Vibration Bands
PU_0	PU_1	PU_2	PU_3	PU_4	PU_5	PU_6	PU_7	PU_8	PU_9	PU_10	PU_11	PU_12	Sample
3320	3320	3319	3321	3320	3321	3321	3321	3321	3321	3319	3320	3321	H-bonded N–H stretching
2936	2936	2936	2936	2936	2935	2936	2935	2935	2937	2937	2938	2938	CH_2_ asymmetric
2864	2864	2863	2864	2864	2864	2864	2864	2864	2864	2864	2864	2864	CH_2_ symmetric
1738	1738	1736	1738	1737	1738	1740	1739	1738	1739	1739	1740	1738	C=O non-hydrogen-bonded (ester) stretching
1728	1727	1726	1728	1727	1727	1727	1728	1727	1728	1727	1726	1727	C=O hydrogen bonded in HS–SS (ester) stretching
1711	1709	1707	1710	1710	1708	1708	1711	1708	1709	1707	1705	1705	C=O not hydrogen-bonded (urethane) stretching
1684	1685	1685	1686	1686	1684	1684	1685	1684	1683	1683	1683	1683	C=O hydrogen bonded in HS–HS (urethane)
1535	1534	1535	1535	1535	1533	1536	1533	1533	1537	1536	1536	1536	N–H deformation
1160	1160	1159	1160	1159	1161	1161	1161	1161	1160	1159	1160	1169	C–O–C stretching
1094	1098	1098	1099	1099	1099	1105	1099	1099	1105	1108	1104	1106	
1039	1043	1041	1042	1042	1043	1042	1043	1043	1033	1042	1042	1044	

**Table 3:** Hydrogen bond index (R) and degree of phase separation (DPS) for PU and PU modified with PCMs.

Sample	Hydrogen Bond Index (R)	Degree of Phase Separation (DPS)
PU_0	0.674	0.402
PU_1	0.342	0.255
PU_2	0.543	0.352
PU_3	0.533	0.348
PU_4	0.576	0.365
PU_5	0.619	0.382
PU_6	0.437	0.304
PU_7	0.426	0.299
PU_8	0.503	0.335
PU_9	0.658	0.397
PU_10	0.647	0.393
PU_11	0.726	0.421
PU_12	0.409	0.291

**Table 4:** Setting times of selected PU/PCM systems.

Sample	Curing Time of PU	T_max_ [°C]
PU_0	14 min 45 s	55.1
PU_1	16 min	49.1
PU_3	14 min 15 s	49.0
PU_6	16 min 30 s	47.9
PU_10	20 min 15 s	47.5

## Data Availability

The authors confirm that the data supporting the findings of this study are available within the article.
